# Localized Pancreatitis in an Elderly Patient Without Suspected Etiology

**DOI:** 10.7759/cureus.28034

**Published:** 2022-08-15

**Authors:** Yutaro Yamamoto, Ryuichi Ohta, Yudai Tanaka, Go Mishiro, Chiaki Sano

**Affiliations:** 1 Family Medicine, Shimane University Faculty of Medicine, Izumo, JPN; 2 Community Care, Unnan City Hospital, Unnan, JPN; 3 Internal Medicine, Unnan City Hospital, Unnan, JPN; 4 Community Medicine Management, Shimane University Faculty of Medicine, Izumo, JPN

**Keywords:** pancreatitis, localized pancreatitis, community hospitals, japan, rural, metabolic and anatomical changes, super-aged patient, acute pancreatitis

## Abstract

Once pancreatic inflammation is triggered, it spreads throughout the pancreas. Here, we present a case of localized pancreatitis wherein the inflammation was confined to the pancreatic head. A 91-year-old woman was admitted with complaints of vomiting and epigastric pain. Blood tests showed elevated pancreatic enzyme levels, whereas imaging studies showed an enlarged pancreatic head and an area of increased density in the surrounding fatty tissue extending along the retroperitoneum below the subrenal pole. Atrophy of the pancreatic parenchyma in the pancreatic body and tail and dilatation of the main pancreatic duct were observed. The patient was diagnosed with acute pancreatitis, was kept nil by mouth, and was administered supplemental fluids. The symptoms resolved within two weeks. Age-related anatomical and histological changes in the pancreas may influence the development of pancreatitis, making it difficult to rule out the possibility of cancer. As age-related changes in the pancreas could lead to the development of pancreatitis, it is an important differential diagnosis of abdominal pain, even in older patients without suspected etiologies.

## Introduction

Pancreatitis is a critical disease in which pancreatic enzymes, such as lipase and amylase, cause severe abdominal inflammation [1,2]. Inflammation in other organs damages multiple organs, manifesting as severe peritonitis and liver and renal failure [3]. The mortality rate is high in cases requiring advanced medical care, reaching 4-10% [3]. However, definitive treatments for pancreatitis are lacking; therefore, supportive care alone is required [4]. Diagnostic accuracy, adequate hydration, appropriate nutrition, and etiology determination are essential for improving patients' mortality and morbidity rates with pancreatitis [4].

Aging has changed the prevalence of etiologies of pancreatitis. The main etiologies of pancreatitis are cholelithiasis, alcohol consumption, and various infections [5]. However, few older people consume large amounts of alcohol [5]. Gallstones can cause pancreatitis in older patients, but there are various causes of pancreatitis unrelated to alcohol consumption or gallstones [6]. These cases could be attributed to idiopathic or anatomical abnormalities of the cholangial and pancreatic ducts and duodenal diverticulum [7]. These causes can be demonstrated by advanced imaging tests, such as magnetic resonance imaging (MRI), magnetic resonance cholangiopancreatography (MRCP), and other endoscopic procedures [5]. However, invasive tests cannot always be performed in older adults because of their limitations in performing activities of daily living and their tendency to avoid invasive tests due to co-morbidities, especially cardiac risk.

Furthermore, pancreatitis in older patients can cause different inflammatory patterns on imaging tests because of various age-related changes in anatomy and physiology [1,2]. Generally, pancreatitis occurs throughout the organ because it triggers inflammation and causes its spread [2]. In the present case, we observed localized pancreatitis in the pancreatic head, which may have been caused by various anatomical variations and pre-existing patient conditions. In this case report, we present a rare case of acute pancreatitis in an older patient and discuss the pathophysiology causing this rare presentation.

## Case presentation

A 91-year-old woman with level 2 care requirement, who lived in a group home, was brought to our hospital complaining of vomiting and epigastric pain. The day before her arrival, she appeared less responsive than usual. Upon arrival, a group home staff member reported seeing her vomiting in the toilet. The patient complained of epigastric pain; however, her symptoms were not alleviated during the clinical course. The patient was then transferred to our hospital for further investigation. Her medical history included acute pancreatitis treated two years ago, cholecystitis and cholangitis with cholecystectomy five years ago, dementia, hypertension, and dyslipidemia. Her medications included donepezil 5 mg/day, atorvastatin 5 mg/day, and magnesium oxide 660 mg/day.

On presentation, her consciousness was clear, and her vital signs were as follows: blood pressure of 120/72 mmHg, heart rate of 52 beats/minute, respiratory rate of 16 breaths/minute, oxygen saturation of 98% on room air, and body temperature of 36.4°C. Ocular conjunctival pallor or icterus was not observed. Abdominal examination revealed no increase or decrease in bowel peristalsis; however, tenderness, pain on percussion, and abdominal guarding were observed in the upper abdomen to the umbilical area without Cullen’s sign. Blood tests on admission showed an elevated inflammatory response with a white blood cell count of 10.50 × 103/µL, pancreatic amylase level of 2418 IU/L, and lipase level of 8050 IU/L. No obvious elevations in bilirubin, cholesterol, and antinuclear antibody levels, which were measured to investigate the reason for several episodes of pancreatitis, were observed (Table 1).

**Table 1:** Laboratory test results CA19-9, carbohydrate antigen 19-9; CEA, carcinoembryonic antigen; CRP, C-reactive protein; PO2, partial pressure of oxygen.

Marker	Normal range	Day 1	Day 2	Day 4	Day 5
White blood cells, ×10^3^/μL	3.5-9.1	10.50	11.40	11.40	7.60
Platelets, ×10^4^/µL	13.0-36.9	9.2	7.8	6.5	7.5
PO_2_, mmHg	>75	17.9/79.8			
Base excess, mmol/L	-2.4 to 2.3	0.9			
Aspartate aminotransferase, IU/L	8-38	24	23	22	18
Alanine aminotransferase, IU/L	4-43	9	8	7	8
Lactate dehydrogenase, U/L	121-245	203	236	292	227
Serum amylase, IU/L	40-120		657	122	70
Pancreatic amylase, IU/L	18-55	2418			
Lipase, IU/L	13-55	8060	710	79	63
Blood urea nitrogen, mg/dL	8-20	15.8	20.9	13.7	11.2
Serum Ca, mg/dL	8.8-10.1	8.6	8.5	7.9	8.0
CRP, mg/dL	<0.30	0.23			
CEA, ng/mL	<5.0		<1.7		
CA19-9, U/mL	<37.0		24.8		
Antinuclear antibody, n times	<40	<40			

**Figure 1:**
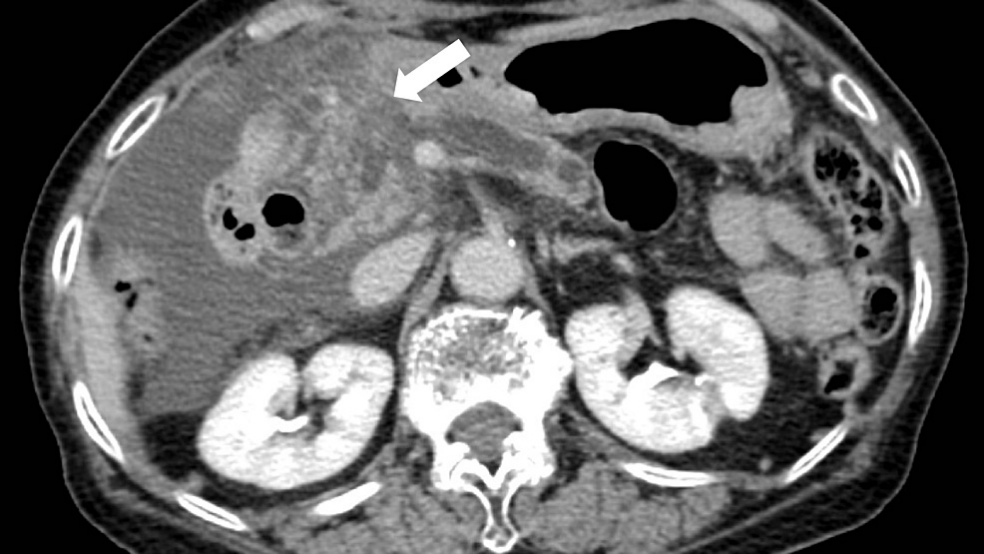
Contrast-enhanced computed tomography image of the abdominopelvic region showing an enlarged pancreatic head (white arrow)

Contrast-enhanced computed tomography (CECT) of the abdominopelvic region showed an enlarged pancreatic head with an area of increased density extending from within the parenchyma to the surrounding fatty tissue and along the retroperitoneum below the subrenal pole. Atrophy of the pancreatic parenchyma in the caudal pancreatic body and dilatation of the main pancreatic duct were also observed (Figure 1).

**Figure 2:**
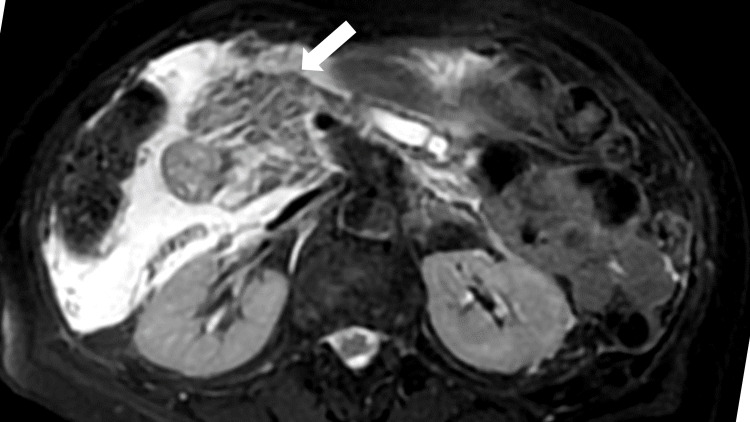
Magnetic resonance image showing an enlarged pancreatic head (white arrow)

MRI and MRCP revealed an enlarged pancreatic head with a mildly increased signal on T2-weighted imaging (T2WI). The surrounding fatty tissues showed extensive T2WI high-signal areas. The main pancreatic duct was dilated, with interrupted dilatation of the pancreatic head and body. Atrophy was observed in the pancreatic parenchyma within the caudal portion of the pancreatic body (Figure 2).

The patient was diagnosed with acute pancreatitis localized to the head of the pancreas and was managed with fasting and supplemental fluids during the hospital stay.

The pancreatic tumor markers were not elevated, with levels of the carcinoembryonic antigen of <1.7 ng/mL and carbohydrate antigen 19-9 (CA19-9) of 24.8 U/mL. The pancreatic enzyme levels were significantly reduced by the fourth day after admission, with an overall trend toward improvement (Table 1). After admission, the patient was treated with fasting and supplemental fluids until the third day of admission. On the fourth day, considering the improvement in her general condition and laboratory values, she resumed oral intake of a low-fat diet with reduced fluid volume.

**Figure 3:**
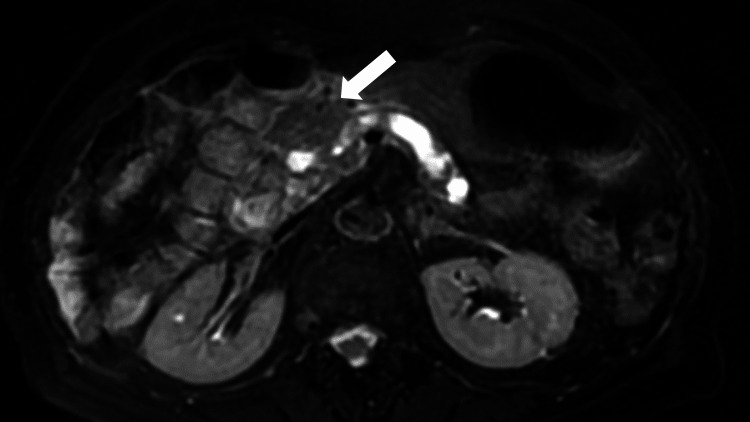
Magnetic resonance image showing a decreasing trend of findings of acute pancreatitis in the pancreatic head with mild swelling in the pancreatic head (white arrow)

On the fifth day after admission, the patient developed a fever of 38.6°C with infiltration of the right lung on a chest X-ray. Considering the possibility of aspiration pneumonia and infection of the pancreatic head, she was treated with fasting and supplemental fluids and the antibacterial drug sulbactam/ampicillin 6.0 g/day for 10 days. Based on her general condition and trend toward fever resolution, a low-fat diet was resumed on the sixth day after admission. Physical examination of the abdomen revealed persistent symptoms of peritoneal irritation and tenderness that disappeared on the 12th day after admission. MRI/MRCP showed a decrease in the findings of acute pancreatitis in the pancreatic head with mild swelling and mildly increased signaling on T2WI but no obvious tumor lesions (Figure 3).

The patient had a good clinical course and was discharged home on the 15th day after admission.

## Discussion

Our case report shows that the presentation of older patients with acute pancreatitis could be unusual because of accumulated age-related changes. In contrast, the pathophysiology of the rare presentation could be affected by various metabolic and anatomical changes.

Diverse factors are relevant to the pathogenesis of idiopathic pancreatitis localized to the pancreatic head in elderly individuals. Age-related atrophy of the pancreatic tissue may result in atrophy of the pancreatic parenchyma, which may slow the spread of inflammation due to pancreatitis compared to younger patients [7]. The site of atrophy may also affect the extent of inflammation [8]. In the present case, the patient had previously undergone pancreatic duct dilatation, and atrophy was observed from the pancreatic body to the tail. Therefore, the pancreatic head, which showed relatively little atrophy, may have been strongly inflamed and observed as a tumor. Diverticulum formation in the duodenum next to the ampulla of Vater can cause pancreatic duct obstruction, which can lead to pancreatitis [9]. The frequency of diverticula reportedly increases with age; in this case, diverticulum formation around the peripapillary region was also observed on imaging [9]. In present epidemiology, alcohol and gallstones are still the major causes of pancreatitis. However, as the population ages, the causes of pancreatitis have become more diverse. Clinicians need to identify pancreatitis in the future in the context of abdominal pain in elderly patients.

In addition to a close examination of the common causes of pancreatitis in the elderly, it is necessary to evaluate the presence of alcohol consumption and gallstones, as well as the presence or absence of autoimmunity, original pancreatic duct dilatation, and diverticula [7,9]. The incidence of pancreatitis in the elderly gradually increases, and an accurate and prompt diagnosis determines its prognosis [8]. A history of gallstones and alcohol consumption is significant. Alcohol consumption and gallstones are common causes of pancreatitis in older people, with increasing alcohol consumption and obesity among the elderly population owing to the increase in healthy life expectancy [10]. However, attention should also be paid to anatomical abnormalities of the pancreas and surrounding organs. Various medical conditions, such as in our case, can cause pancreatitis. Age-related anatomical changes in the pancreas may influence the development of pancreatitis [11]. It is necessary to check the patient’s past imaging and history constantly; if bile duct or pancreatic duct dilatation is noted or there is a history of similar abdominal pain, MRCP and hepatobiliary scrutiny should be performed aggressively.

Appropriate history and physical examination of elderly individuals are important diagnostic strategies in community hospitals. Pancreatitis may be overlooked in older patients with vague symptoms [12,13]. In response to abdominal pain symptoms in the elderly, appropriate medical history, physical examination, and non-invasive abdominal ultrasound should be performed in community hospitals to identify and investigate the possibility of pancreatitis [5]. Furthermore, elderly people often self-manage their symptoms, which may exacerbate them [14,15]. To promote appropriate diagnosis and treatment of pancreatitis in the future, it is necessary to accumulate relevant information on pancreatitis in the community and educate people about the importance of appropriate self-management and timely consultation regarding their symptoms [16].

## Conclusions

The patient was very old and presented with acute and benign pancreatic head enlargement. This case suggests that age-related anatomical changes in the pancreas may influence the development of pancreatitis and that such etiologies should be considered in older patients with a past medical history of pancreatitis. In a community hospital setting, appropriate history taking, physical examination, and non-invasive abdominal ultrasound can identify and investigate pancreatitis in elderly patients despite vague symptoms.
